# Antioxidant Metabolism Pathways in Vitamins, Polyphenols, and Selenium: Parallels and Divergences

**DOI:** 10.3390/ijms25052600

**Published:** 2024-02-23

**Authors:** Celia María Curieses Andrés, José Manuel Pérez de la Lastra, Celia Andrés Juan, Francisco J. Plou, Eduardo Pérez-Lebeña

**Affiliations:** 1 Hospital Clínico Universitario, Avenida de Ramón y Cajal, 3, 47003 Valladolid, Spain; cmcurieses@gmail.com; 2 Institute of Natural Products and Agrobiology, CSIC-Spanish Research Council, Avda. Astrofísico Fco. Sánchez, 3, 38206 La Laguna, Spain; 3 Cinquima Institute and Department of Organic Chemistry, Faculty of Sciences, Valladolid University, Paseo de Belén, 7, 47011 Valladolid, Spain; celia.andres.juan@uva.es; 4 Institute of Catalysis and Petrochemistry, CSIC-Spanish Research Council, 28049 Madrid, Spain; fplou@icp.csic.es; 5 Sistemas de Biotecnología y Recursos Naturales, 47625 Valladolid, Spain; info@glize.eu

**Keywords:** antioxidants, diseases, vitamins, polyphenols, human diet

## Abstract

Free radicals (FRs) are unstable molecules that cause reactive stress (RS), an imbalance between reactive oxygen and nitrogen species in the body and its ability to neutralize them. These species are generated by both internal and external factors and can damage cellular lipids, proteins, and DNA. Antioxidants prevent or slow down the oxidation process by interrupting the transfer of electrons between substances and reactive agents. This is particularly important at the cellular level because oxidation reactions lead to the formation of FR and contribute to various diseases. As we age, RS accumulates and leads to organ dysfunction and age-related disorders. Polyphenols; vitamins A, C, and E; and selenoproteins possess antioxidant properties and may have a role in preventing and treating certain human diseases associated with RS. In this review, we explore the current evidence on the potential benefits of dietary supplementation and investigate the intricate connection between SIRT1, a crucial regulator of aging and longevity; the transcription factor NRF2; and polyphenols, vitamins, and selenium. Finally, we discuss the positive effects of antioxidant molecules, such as reducing RS, and their potential in slowing down several diseases.

## 1. Introduction

Metabolic processes in cell mammals, such as respiration and digestion, produce millions of free radicals (FRs) every day [1]. Our body has its own natural antioxidant mechanisms, and the intake of dietary antioxidants enhances their effectiveness, along with the inherent antioxidant properties of the food [2].

Some substances increase oxidative processes in our bodies, leading to accelerated aging and disease development, such as pollution, smoking, excessive alcohol consumption, high-fat diets, intense exercise, prolonged exposure to sunlight, and chronic stress. Antioxidants like vitamins A, C, and E; β-carotene; polyphenols; selenium; zinc; and copper can counteract the harmful effects of FR [3]. According to Giuseppe Murdaca, vitamin D plays a key role in calcium homeostasis and possesses antioxidant, anti-inflammatory, immunomodulatory, and anti-fibrotic activities [4].

The Mediterranean diet emphasizes the consumption of vegetables and fruits as rich sources of antioxidants [5]. Evidence supports the notion that a diet rich in vegetables and fruits promotes overall health and reduces the risk of certain diseases [6].

Oxidative stress (OS) is implicated in the development of human diseases [7], and researchers in pharmacology are actively investigating the use of antioxidants for the prevention and treatment of stroke and neurodegenerative disorders [8]. Antioxidant supplements have gained popularity among individuals aiming to maintain good health and prevent cancer and heart disease [9]. Some studies suggest that their benefits and large-scale clinical trials have not consistently found positive effects, and excessive intake may even be harmful. Antioxidants are not only used in medicine, but also in various industries as preservatives in food and cosmetics, preventing the degradation of rubber, polymers, and gasoline [10].

The novelty of this contribution lies in the comprehensive examination of the similarities and differences between the metabolic pathways of vitamins, polyphenols, and selenium, three major classes of antioxidants. While both play crucial roles in protecting cells from oxidative damage, their molecular structures and metabolic fates differ significantly. Here, we have attempted to provide an overview of these similarities and differences, highlighting the metabolic characteristics of each type of antioxidant, including the transcriptional and enzymatic regulation of the three types of compounds. By exploring the metabolism pathways of polyphenols, vitamins, and selenium (Se) in the body, this manuscript contributes to a deeper understanding of their potential health benefits.

## 2. Relationship between RS, Aging, and Related Diseases

Reactive oxygen, nitrogen, and halogen species, collectively referred to as reactive species (RS), are produced through various internal and external processes. These species have harmful effects that need to be counteracted by antioxidant pathways. An imbalance between this process and the antioxidant defense leads to stress on cellular proteins, lipids, and DNA [11,12]. Aging is characterized by the progressive deterioration of tissues and organs. The accumulation of RS contributes to functional decline and age-related diseases such as cardiovascular diseases, neurodegenerative disorders, and cancer [13].

FR are reactive molecules with unpaired electrons in their outer shells. Reactive oxygen species (ROS) and reactive nitrogen species (RNS) are types of FR produced in aerobic cells, playing a role in aging and age-related diseases [14]. RS induces cellular senescence, which hampers cell reproduction and proliferation in response to damage, and senescent cells develop a phenotype characterized by the secretion of factors like interleukins, chemokines, matrix metalloproteases (MMPs), and extracellular matrix (ECM) proteins [15].

RS induce senescence through various mechanisms, including: (i) regulation of mammalian target complexes of rapamycin; (ii) production of pro-inflammatory interleukins, such as IL-1α, which stimulate factors like nuclear factor kappa-B (NF-κB); (iii) triggering chronic diseases such as cancer, Alzheimer’s disease, atherosclerosis, osteoarthritis, and emphysema; (iv) inhibiting forkhead box (FOXO) proteins involved in protection against OS; and (v) inhibiting the activity of sirtuin and SOD enzymes, thereby increasing OS and promoting a pro-inflammatory state [16,17].

Researchers have identified biomarkers that provide valuable information about its progression and the effectiveness of potential treatments, and these biomarkers aid in the selection of drugs that can attenuate or modulate RS by targeting specific therapeutic pathways [18].

RS play a crucial role in aging and the development of various clinical diseases, suggesting that antioxidant therapy may positively impact disease progression. However, further research is necessary to assess the true efficacy of these potential therapeutic interventions [19].

## 3. Molecular Antioxidant Capacity and Antioxidant Defenses

### 3.1. Definition and Features of Antioxidant Capacity

Antioxidants have the ability to slow down or prevent the oxidation of other molecules, and their consumption has gained attention in the medical field for the treatment of various diseases, significantly delaying the oxidation of cellular components [20] and protecting cells from damage caused by FRs. FRs are unstable molecules produced during normal physiological or pathological metabolic processes and are known to contribute to the development of cancer, heart disease, stroke, diabetes, and age-related conditions [21]. Antioxidants essentially act as scavengers, neutralizing FRs [22]. At present, there are no values with which to assess antioxidants in food labeling, but it would be desirable to establish a standardized method to measure the total antioxidant capacity (TAC) of plant extracts or foods [23].

The antioxidant capacity depends on several properties: (i) the presence of reducing substituents [24]; (ii) the chelating ability of transition metals, determined by functional groups in the molecule [25]; (iii) the accessibility and bioavailability of the antioxidant (Niki, 1999); and (iv) the interaction between oxidant radicals and antioxidant compounds [26].

Antioxidants can be classified as water-soluble (they interact in the cytoplasms of cells and in blood plasma) or fat-soluble, protecting cell membranes from lipid peroxidation [27]. Antioxidants can be exogenous (obtained from dietary intake) or endogenous, synthesized by our metabolism [21].

### 3.2. Antioxidant Defenses

**Figure 1:**
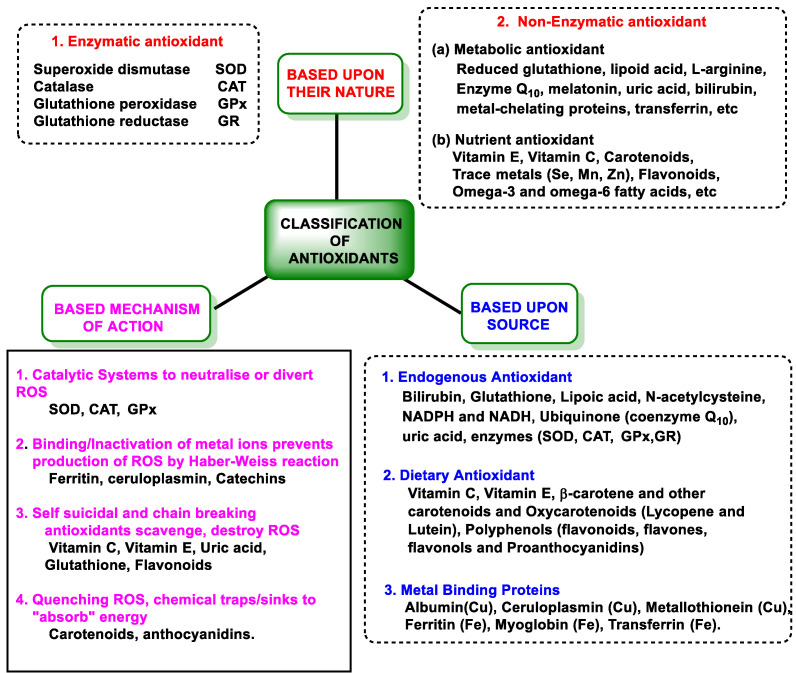
Classification of antioxidants.

Figure 1 shows a classification according to the type, pathway, and origin of antioxidants.

#### 3.2.1. Endogenous Defenses

Endogenous antioxidant defense includes enzymatic and non-enzymatic mechanisms [28]. The endogenous pathway consists of SOD, which detoxifies superoxide anion (^•^O_2_^−^); catalase (CAT) and glutathione peroxidase (GPx), which are involved in the detoxification of peroxides; and CAT against H_2_O_2_ and GPx, which catalyzes the reduction of H_2_O_2_ or organic hydroperoxides to water or corresponding alcohols by glutathione GSH, thus playing a central role in the mammalian antioxidant system [29].

Glutathione reductase (GR) is involved in the regeneration of oxidized glutathione; thioredoxin reductase (TrxR) in the protection against protein oxidation; and glucose-6-phosphate dehydrogenase (G6PD) in the regeneration of NADPH [30].

**Figure 2:**
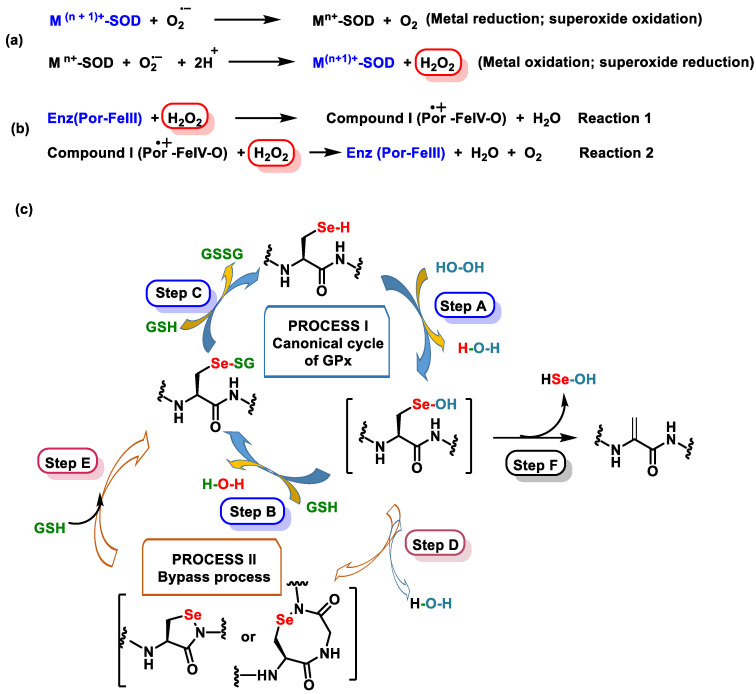
(**a**) SOD-catalyzed dismutation of ^•^O_2_^−^. M = [Cu (n = 1); Mn and Fe (n = 2)]. The oxidation state of metal cation varies between n and n + 1. (**b**) CAT-catalyzed mechanism of H_2_O_2_ dismutation. (**c**) Catalytic cycle of GPx for H_2_O_2_ reduction. Step A: Selenol (-SeH) in GPX is oxidized to selenic acid (-Se-OH) by H_2_O_2_. Step B: The first GSH molecule reduces selenic acid (-Se-OH) to form glutathioneated selenol intermediate (-Se-SG) and releases a part of H_2_O. Step C: The second GSH molecule continues to reduce the intermediate (Se-SG) to form oxidized glutathione (GSSG), while the activity of GPX neutrality returns to selenol (-SeH [32,33]). (Process II) bypass mechanism. Flohé et al. proposed [34] that Sec–SeOHs could undergo intramolecular cyclization to the corresponding cyclic selenenyl amides, with either a five-membered ring or an eight-membered ring (Step D), to prevent thermal deselenation (Step F) under GSH-deficient conditions.

Non-enzymatic pathways are controlled by glutathione (GSH, ubiquitous in cells), uric acid, lipoic acid, NADPH, coenzyme Q, albumin, bilirubin, etc. [31]. SOD-catalyzed dismutation of the ^•^O_2_^−^ is characterized in Figure 2, as well as the catalytic mechanism catalyzed by CAT for the reduction of H_2_O_2_ to H_2_O and O_2_ [29].

H_2_O_2_ can be directly removed by the CAT enzyme, producing O_2_ and H_2_O, while GPx uses H_2_O_2_ and reduced glutathione GSH to form H_2_O and oxidized glutathione GSSG. In the Fenton reaction, ferrous iron Fe^2+^ reacts with H_2_O_2_, generating Fe^3+^, OH^−^, and ^•^OH [31,35].

The intake of foods rich in natural antioxidants is recommended by health organizations [36]. Since humans cannot synthesize these antioxidant compounds ex novo, plant-based foods such as apples, plums, bananas, tomatoes, potatoes, onions, broccoli, and others serve as primary sources of these antioxidants [37]. Synthetic additives such as butylated hydroxyanisole (BHA); butylated hydroxytoluene (BHT); tert-butylhydroquinone (TBHQ); and propyl, octyl, and dodecyl gallates preserve the shelf lives of processed foods [38].

#### 3.2.2. Vitamins and Polyphenols as Exogenous Antioxidants

Exogenous antioxidants are vitamins A, C, and E (α- and γ-tocopherol); carotenoids; and polyphenols (flavonoids, tannins, phenols, and lignans) [39,40]. Diet is the main source of exogenous antioxidants, and supplementation is becoming increasingly important [41]. Due to the current lower consumption of fruits and vegetables, it may be difficult to acquire sufficient exogenous antioxidants, so ensuring this intake is essential to the redox balance in cellular homeostasis [42].

Vitamin A (Vit A) consists of unsaturated organic nutrient compounds (including retinol, retinal, and retinoic acid) and various provitamin A carotenoids (such as β-carotene). Food sources of Vit A include foie gras, pâtés, margarine, butter, cheese, chard, carrots, kohlrabi, spinach, tomatoes, persimmons, apricots, melons, lettuce, leeks, mangoes, plums, peaches, squash, zucchini, asparagus, eggs, oysters, herring, sardines, clams, and more. Vit A’s functions include growth and development, the immune system, and vision [43]. Globally, about one-third of children under five suffer from Vit A deficiency. This is estimated to cause 670,000 deaths annually in this age group, and is also a leading cause of childhood blindness, which is most prevalent in Southeast Asia and Africa [44,45]. Its antioxidant effect is attributed to its hydrophobic polyene chain, neutralizing singlet oxygen and thiyl radicals and stabilizing peroxyl radicals [46]. Vit E influences the differentiation and proliferation of immune system T-cells through an indirect mechanism in interleukin IL-2 [47].

**Figure 3:**
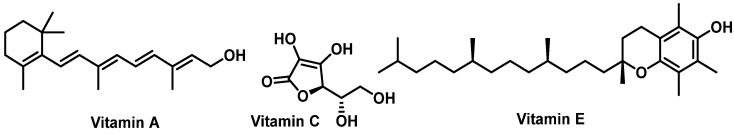
Structure of vitamins A, C, and E.

Vitamin C (Vit C, ascorbic acid, or ascorbate), is present in citrus fruits and vegetables [48], and is available as a dietary supplement and in topical serums for the treatment of melasma and facial wrinkles [49]. It is essential for preventing and treating scurvy, with roles in tissue repair, collagen formation, enzymatic production, and survival [50]. It is a water-soluble electron donor [51], and several epidemiological studies have demonstrated that a diet rich in fruits and vegetables is associated with a lower risk of cardiovascular disease CVD, stroke, cancer, and increased life expectancy. The antioxidant effect of dietary sources is not exclusively due to Vit C [52], and studies on healthy individuals have indicated a sigmoidal relationship between oral dose and plasma and tissue concentrations of Vit C. The molecular structures of vitamins A, E, and C are depicted in Figure 3.

Obtaining vitamin E (Vit E) through food intake is not a problem, as breakfast cereals and fruit juices are fortified [53]. Several natural food sources are particularly rich in Vit E (wheat germ oil, almonds, sunflower seeds, pine nuts, avocado, peanut butter, fish, and red peppers [54]). The absorption of Vit E by the digestive system requires some fat. Its deficiency is extremely uncommon among healthy individuals, but is associated with certain diseases that impair the digestion or absorption of fat. Such conditions include Crohn’s disease, cystic fibrosis, and rare genetic disorders like abetalipoproteinemia and ataxia with Vit E deficiency (AVED). Vit E deficiency can result in nerve and muscle damage, with a loss of sensation in the arms and legs, impaired motor control, muscle weakness, visual disturbances, and a weakened immune system [55]. Vit E’s structure is a methylated phenolic compound with four tocopherols and four tocotrienols, α-tocopherol being the most common type and γ-tocopherol being the most common form in the American diet [56]. Tocopherols contain an aromatic ring with a hydroxyl that can donate H^+^ to reduce FRs and a hydrophobic side chain able to penetrate into biological membranes [57] (Table 1).

Polyphenols are a large family of natural compounds characterized by the presence of phenolic hydroxyls (with aromatic rings), which include four main classes: phenolic acids, flavonoids, stilbenes, and lignans [58].

Proanthocyanidins are macromolecules with a molecular weight of more than 200 Daltons, with rapid diffusion across cell membranes [59]. Larger polyphenols are biosynthesized in situ from smaller polyphenols to non-hydrolysable tannins [60]. Some polyphenols contain repeating phenolic molecules of pyrocatechin, resorcinol, pyrogallol, and chloroglucinol, linked by esters (hydrolysable tannins) or by more stable C-C bonds [61].

Dietary polyphenols have received enormous attention from scientists and nutritionists due to their well-known role in human health, because they can help to prevent degenerative diseases such as cancer, CVD, and neurological disorders [62], and their therapeutic effects are based on the regulation of cellular signaling pathways [63,64].

**Figure 4:**
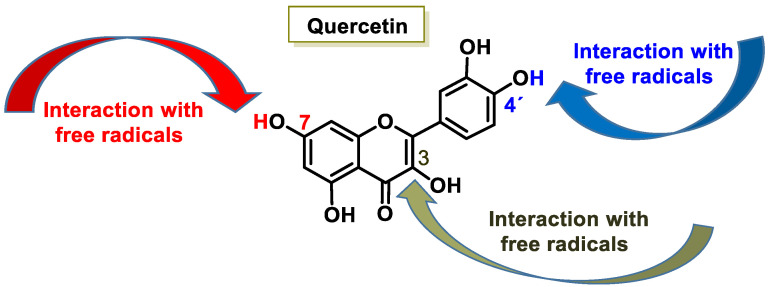
Alkoxy radical positions at C-7, C-3, and C-4′.

**Figure 5:**
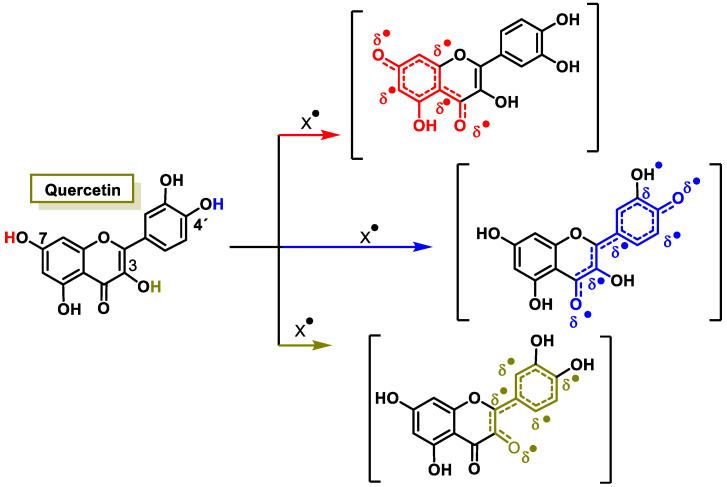
For quercetin, electron delocalization from C-7, C-3, and C-4′ positions.

Their antioxidant effect is based on the ability of the aromatic hydroxyl groups to donate an H^+^ to FRs, such as hydroxyl, peroxyl, etc., which lose reactivity by forming a relatively stable flavonoid radical [65]. Figure 4 and Figure 5, for quercetin, show the biochemical mechanism of FR uptake according to the thesis proposed by Perez de la Lastra et al., 2022 [66]. The ^•^X radical can be an oxygen, nitrogen, or chlorine radical such as hydroxyl, peroxyl, ^•^O_2_^−^, or peroxynitrous acid.

The interaction of an FR (^•^X) at the OH of the C-7 position resulted in electron delocalization throughout the A-ring (Figure 5). When ^•^X was generated at C-3 and C-4′ OH positions of quercetin, there was a greater delocalization of the unpaired electron, yielding a greater number of resonant forms (in blue and green). OH located at the 3 and 4′ positions was predicted to have greater antioxidant activity.

In addition to scavenging radicals, polyphenols also act as metal chelators [67]. Chelation of Fe^2+^ can directly reduce the rate of the Fenton reaction, thus preventing oxidation caused by highly reactive OH radicals [68,69], and may be involved in the regeneration of antioxidant enzymes [70]. Their antioxidant contribution is generally higher than that of vitamins A, C, and E [71]. This activity of polyphenols results from a combination of iron chelating and radical scavenging properties, but also refers to the inhibition of lipoxygenase (LO) [72], cyclooxygenase (COX) [73], myeloperoxidase (MPO) [74], NADPH oxidase [75], and xanthine oxidase (XO) [76], preventing the generation of ROS [77] as well as organic hydroperoxides [78]. They also inhibit enzymes indirectly involved in oxidative processes, such as phospholipase A2 (FLA2) [79], while stimulating others with recognized antioxidant properties, such as CAT and SOD [80]. Polyphenols interfere with FR propagation reactions and the formation of the radical itself [81].

Their antioxidant potential depends on the OH group due to their ability to donate H^+^ [82] and capture unpaired electrons via the π-electron system [83]. The higher the electron uptake capacity, the more effective it is as an antioxidant, as the OH groups donate a proton to the hydroxyl, peroxyl, and peroxynitrite radicals, which lose their reactivity due to stabilization and form a relatively stable flavonoid radical [66].

Polyphenols inhibit lipid peroxidation, but they also activate antioxidant enzymes that avoid DNA degradation and prevent the oxidation of the low-density lipoproteins (LDLs) [84]. Indirectly, they (i) increase the activity of red blood cells and the amount of O_2_ reaching the tissues [85]; (ii) have the ability to chelate metal ions such as iron and copper, inhibiting peroxidation reactions of fatty acids and phospholipids in cell membranes [86]; (iii) induce the synthesis of glutathione, increasing the availability of cysteine and avoiding cell membrane peroxidation [87]; (iv) reduce aspartate aminotransferase (AST), alanine aminotransferase (ALT), and fatty acid peroxidation [88]; (v) maintain an optimal redox balance in the cell, which is of particular importance in the mitochondria, where more ROS are generated [89]; (vi) inhibit oxidative enzymes such as XO and NADPH oxidase [90]; and (vii) counteract the oxidative damage induced by H_2_O_2_ in red blood cells, prevent morphological changes, and restore altered functional parameters [91].

**Figure 6:**
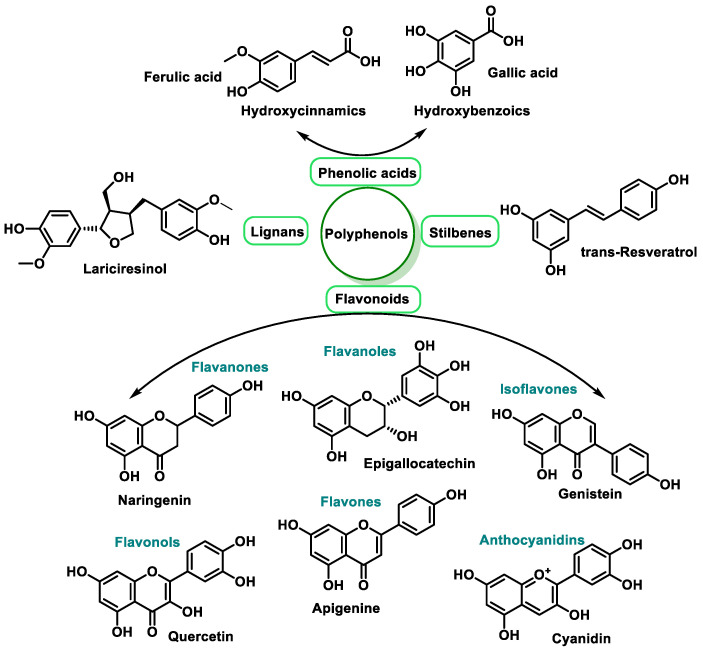
Polyphenol and flavonoid classification.

In summary, the basic feature of polyphenols is based on antioxidant and RF uptake activity, which is even more important than the anti-inflammatory action [92]. A classification of polyphenols is given in the following diagram, Figure 6.

**Figure 7:**
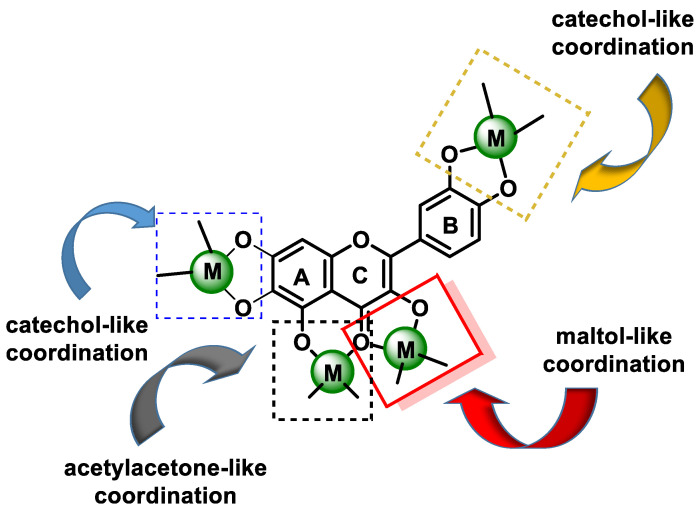
The possible coordination modes of the flavonoid molecules.

Another mechanism that may contribute to the antioxidant activity of phenolic compounds is their ability to chelate redox active metal ions, such as iron, cobalt, manganese, or copper, thus preventing reactions catalyzed by these ions that lead to the formation of oxidative species that can generate oxidative damage at different cellular levels. Fe^2+^ catalyzes, in the presence of hydrogen peroxide, the formation of the hydroxyl radical OH^•^ by means of the Fenton reaction, while the reaction of Cu^+^ with H_2_O_2_ leads to the formation of OH^•^ and O_2_^•−^ radicals [86]. Miličević et al. described a strong correlation between the antioxidant activity of polyphenols and their affinity for Fe(II) ions, suggesting that the suppression of the Fenton reaction is probably due to ion chelation [93]. Interactions of flavonoids with metal ions can lead to the formation of chelates. In the case of flavonoids (flavones), chelating complexes with divalent cations [68,94] can form between:

(a) The 3-hydroxy-4-ketone groups on the C-ring (denoted “site 3-4”), resulting in a maltol-like coordination mode;
(b) The 5-hydroxy group on the A-ring and the 4-carbonyl group on the C-ring (denoted “site 4-5”), resulting in a coordination mode similar to acetylacetone;
(c) 3′,4′-dihydroxy groups located on the B-ring (denoted “3′-4′ site”), resulting in a coordination similar to the catechol mode;
(d) The 6,7-dihydroxy groups on ring A (Figure 7).

**Figure 8:**
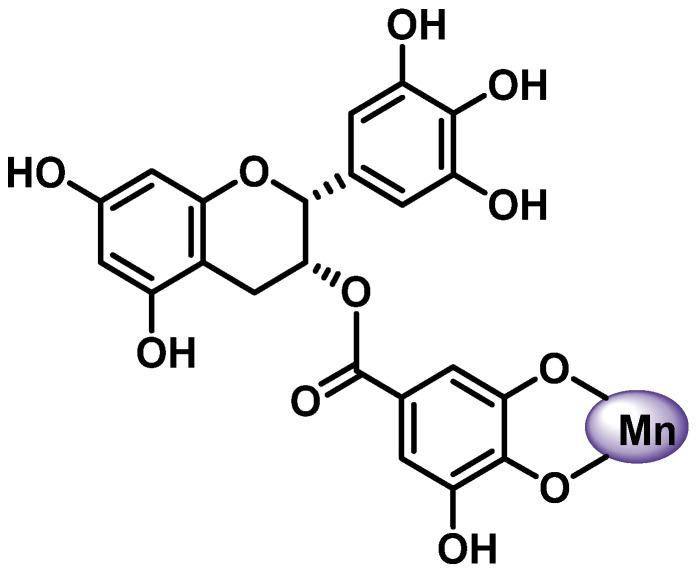
The D-ring of EGCG can form a diolate chelate ring with Mn(II).

In the case of flavonoid glycosides, the hydroxyl groups belonging to the sugar moiety can also participate in metal binding [95]. It has been shown that the two rings, B and D, in the EGCG structure have the same local structure and can participate in metal complexation; however, the OH groups of the D ring represent the preferred coordination sphere around a metal ion [96] (Figure 8).

Metal chelation depends on pH, solvent (polarity and ionic composition), and stoichiometry (ratio of flavonoids to iron). Rutin and negleteine are active inhibitors of the Fenton reaction at very low ratios, while they are prooxidant or ineffective in the vicinity of a 1:1 ratio [97].

Kostyuk et al., 2004, found that metal complexes of rutin, taxifolin, epicatechin, and luteolin with Fe(II), Fe(III), and Cu(II) ions have higher antioxidant activity than free flavonoids [98]. In general, all complexed flavonoids were found to be significantly less oxidized than free flavonoids.

Chelation capacity can be influenced by the reducing properties. For example, Mira et al., 2002, observed that myricetin and quercetin (flavonols with significant reducing activity) had a strong affinity for Fe(III) ions [99].

Structural features influence the complexing ability of flavonoids, but most important are the number and position of hydroxyl groups. Flavonoids with the 6,7-dihydroxy pattern exhibit strong complexation ability at neutral and acidic pH levels. Flavonols with the 3-hydroxyl group, the 4-keto group, and the 2,3-double bond with the catecholic B-ring are strong chelators at neutral and slightly acidic pH levels, and flavonoids with the 5-hydroxyl-4-keto chelation site are weaker chelators even at neutral pH. Several complexes (1:1, 2:1, 1:3, and 2:3, Fe(II):flavonoid) are possible with Fe(II) [100,101]. Quercetin has three possible binding sites for Fe(II) ion chelation. Electrospray ionization mass spectrometry studies have indicated that the preferred flavonoid complexation site is the hydroxyl at carbons 3 or 5 and the adjacent 4-carbonyl group [102].

Electron density is an important factor in the interaction of flavonoids with metal ions. In an aprotic solvent, its influence may be small. In contrast, an aprotic solvent such as water can interact with the phenyl and carbonyl group of a flavonoid, and, depending on the pH, control its dissociation and thus its interaction with metal ions. Flavonoids with higher numbers of hydroxyl groups, such as taxifolin, form complexes with 1:2 and 2:1 stoichiometry at an acidic pH, while a 2:1 complex is observed at neutral and basic pH levels [97].

Therefore, the ability of flavonoids to suppress ROS is mainly based on their chelation of Fe(II) ions, which is influenced by other factors such as pH and the polarity of the reaction medium. On the other hand, Fe(II) ion chelation can significantly influence the properties of flavonoids, such as anti-ROS efficacy, hydrophobicity, and membrane permeability, and, thus, their physiological activity [98,103,104].

The chelating capacity of these flavonoids is associated with potentially beneficial preventive and therapeutic effects, such as neutralization of ROS. The antioxidant properties are mainly exerted through direct free radical scavenging and metal chelation, mainly Fe(II), Fe(III), and Cu(II) [68]. Flavonoid metal complexes have shown greater free radical scavenging properties than the corresponding free flavonoids. In addition, their antitumor activity has been reported to be superior to that of the original flavonoids against several types of cancer cells [105,106].

#### 3.2.3. Role of Selenium in Antioxidant Metabolism

Se is an essential micronutrient and plays a crucial role in metabolism through selenoproteins; these proteins are vital for antioxidant defense and the maintenance of cellular redox balance [107]. Se is a trace element in living organism cells. It is indispensable for metabolism development and holds significant importance for humans, plants, and microorganisms [108].

Physically and chemically, it shares similarities with sulfur, both in its elemental form and in proteins. Sulfur (VI) and Se (VI) oxo-acids tend to oxidize [109].

A deficiency in Se can result in reduced antioxidant protection, impaired immune system function, and various disorders related to the cardiovascular, muscular, endocrine, or neurological systems, with an increased risk of certain cancers, cognitive impairment, and infertility [110].

Inorganic Se is incorporated into the human body through the action of the enzyme GSHPx, which reduces Se to hydrogen selenide (H_2_Se) [111]. Se can be found in its free form in foods that accumulate selenomethionine. Dietary intake of Se is derived from plant products (grains, cereals, fruits, Brazil nuts, broccoli, garlic, onions, and cabbage [112]) which contain selenomethionine and methylselenocysteine. Se from animal sources, as selenocysteine, is obtained from meat, seafood, eggs, and dairy products [113,114].

**Figure 9:**
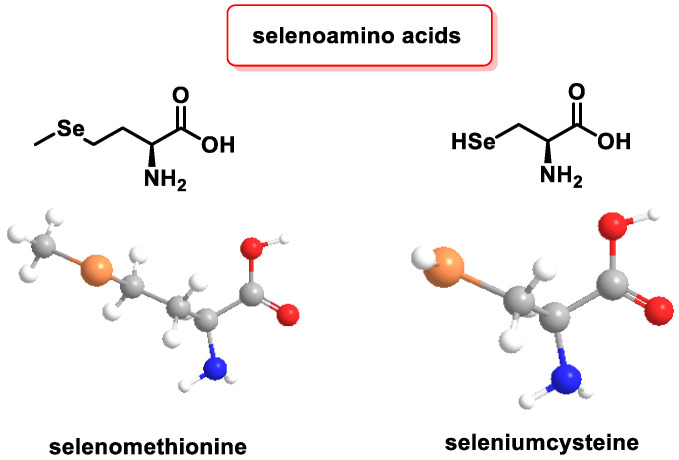
Selenocysteine and selenomethionine chemical structures.

The main organic Se molecules are selenoamino acids (Figure 9), selenopeptides, and selenoproteins.

Selenomethionine (SeMet) is an amino acid analogue of methionine (Met). In SeMet, a sulfur atom in L-Met is substituted or replaced by a Se atom. This modified amino acid is stored in protein reserves and can be randomly incorporated into proteins instead of Met during protein synthesis. When SeMet is catabolized, it releases Se in the form of selenide [115].

Selenocysteine plays a regulatory role in the biological activity of 25 selenoproteins and contributes to the antioxidant, anti-inflammatory, and antiviral properties [116]. Unlike selenomethionine, selenocysteine is not stored, but is directly involved in the catabolism of these proteins. The resulting Se is stored as a reserve for future use [117].

The incorporation of selenocysteine into selenoproteins is a beneficial mechanism for a variety of biological processes [118]. Se exerts its primary biological functions through selenoamino acids, which include: (i) defense against OS, (ii) protection of the cellular redox state and signaling, (iii) participation in the metabolism of lipids, and (iv) involvement in thyroid hormone metabolism [119].

**Figure 10:**
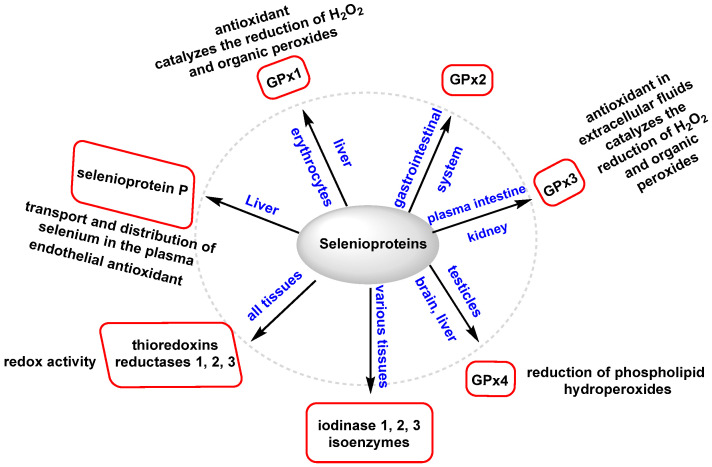
Selenoproteins and their locations.

Se primarily modulates OS through various GPxs and selenoproteins. These enzymes help to reduce the levels of hydrogen peroxide, lipids, and phospholipid hydroperoxides. GPx1 and GPx4 and selenoprotein are abundant selenoproteins involved in these processes [120]. Figure 10 illustrates the main selenoproteins and their respective locations, highlighting the prominent roles of GPx1, GPx4, and selenoprotein P.

Selenium-dependent Gpx catalyzes the H_2_O_2_ and some organic hydroperoxides. GSH is a reducing agent capable of converting H_2_O_2_ and organic hydroperoxides into water. During this process, glutathione itself is oxidized to form oxidized glutathione (GSSG), which in turn is converted to reduced GSH by the enzyme GR [121]. This enzymatic cycle involving GSSG and GR is crucial for maintaining the cellular redox balance and antioxidant defenses. By actively participating in the reduction in ROS and organic hydroperoxides, glutathione plays a vital role in protecting cells from oxidative damage. The coordinated action of GSSG and GR contributes to maintaining the cellular redox state and GSH availability. Selenoprotein is a biomarker for the Se level and also possesses antioxidant properties, characterized by the presence of ten selenocysteine residues, which contribute to its antioxidant function [122].

Selenoprotein P is primarily an extracellular antioxidant, exerting its protective effects by inhibiting the activity of peroxynitrite, a highly reactive oxidant. Additionally, it plays a role in reducing phospholipid hydroperoxides, further contributing to its antioxidant capacity. By engaging in these antioxidant processes, selenoprotein P helps to counteract the harmful effects of OS and maintain the redox balance within the body’s extracellular environment [123].

## 4. Relationship of Antioxidant Metabolism Pathways, Sirtuins, and NRF2

### 4.1. Regulation of Sirtuins SIRT1 and SIRT3

Sirtuins are a class of histone deacetylase proteins, and their enzymatic activity relies on the cofactor NAD^+^. They play a crucial role in various cellular processes, including gene expression, DNA repair, metabolism, mitochondrial function, and biogenesis, and have antioxidant activity. Deregulation of sirtuins has been implicated in the development of diseases like cancer, neurodegeneration, and CVD [124]. Among the sirtuins, SIRT1 is the most extensively studied member. It is associated with insulin sensitivity, tumorigenesis, and the regulation of essential metabolic pathways [125].

SIRT1 provides protection against OS by modulating the acetylation of the FOXO protein. By activating FOXO, SIRT1 increases the expression of antioxidant enzymes like MnSOD and CAT, which counteract the formation of ROS [126,127]. SIRT1 activity is critical for regulating inflammation, and its function can be affected by dynamic fluctuations in the NAD+/NADH ratio during inflammation and OS [128,129].

In recent years, there has been an increasing emphasis on maintaining public health through a balanced diet that includes fruits, vegetables, and antioxidant-rich supplements. Polyphenols, which are abundant in plant-based foods such as fruits, vegetables, tea, cereals, and wine, have been associated with various health benefits [59,130].

Certain small polyphenolic molecules, including resveratrol, fisetin, quercetin, and curcumin, have been found to modulate SIRT1 activity [131]. Resveratrol, for example, has been shown to activate the immune system and extend the lifespan in yeast, worms, and flies [132,133,134].

Quercetin, a flavonol with antioxidant and anti-inflammatory properties, has been studied for its effects on reducing OS, inhibiting LDL oxidation and platelet aggregation, and acting as a vasodilator [135]. Quercetin has also shown promising potential in ameliorating atherosclerosis by inhibiting OS and inflammatory responses through the AMPK/SIRT1/NF-κβ signaling pathway [136,137].

Ilenia Bazzucchi et al., 2020, studied whether quercetin supplementation improves neuromuscular function recovery from muscle damage. The results showed that quercetin supplementation significantly attenuated the strength loss compared to a placebo. Quercetin supplementation for 14 days seems able to ameliorate the recovery from eccentric exercise-induced weakness and neuromuscular function impairment. Biochemical parameters probably increase due to its strong anti-inflammatory and antioxidant action [138]. Paolo Sgrò et al., 2021, studied the effects of quercetin modulation on IGF-I and IGF-II levels after eccentric exercise-induced muscle damage. After supplementation, there was a more marked increase in IGF-I levels, and notably, the IGF-II peak was found earlier compared to the placebo, at the same time as IGF-I. Quercetin significantly reduced plasma markers of cell damage and the interleukin 6 level during the recovery period following EIMD compared to the placebo [139].

Curcumin, a natural bioactive polyphenolic compound, possesses antioxidant, anti-cancer, and anti-inflammatory properties [140]. It scavenges reactive oxygen and nitrogen species, increases the expression of antioxidant proteins, and can activate SIRT1. Curcumin-induced upregulation of SIRT1 has been associated with beneficial effects on various diseases, including cardiac fibrosis, diabetes, and ischemia/reperfusion injury [141,142].

SIRT3 is a histone deacetylase located in the mitochondria that responds to RS and protects cells from genotoxic damage mediated by oxidative processes, thereby reducing cell apoptosis mediated by genotoxins and OS, maintaining mitochondrial homeostasis by deacetylating substrates in an NAD^+^-dependent manner [143]. SIRT3 (widely expressed in mitochondria-rich tissues such as kidney, heart, brain, and liver tissue), is linked to age-related diseases, cancer, and heart and metabolic diseases, suggesting that it may be a potential therapeutic target [144].

Cocoa is rich in polyphenols and has numerous health benefits thanks to its antioxidant properties. Luz del Mar et al., 2023, studied the effect of cocoa polyphenol extract against reactive stress-induced cellular senescence, showing that there was an attenuation of senescent phenotypes and oxidative DNA damage, reducing mitochondrial dysfunction by inhibiting the generation of mitochondrial ROS (mtROS). Cocoa polyphenols induce the expression of SIRT1 and SIRT3 [145].

Wei Wei et al., 2014, explored the protective effects of Vit C and the regulatory mechanism between Vit C and SIRT1. They found that a moderate Vit C concentration of 100 µM prevented cell damage induced by H_2_O_2_, increasing viability, reducing apoptosis, and attenuating intracellular ROS levels, but a higher concentration of Vit C had no effects. Resveratrol is a known activator of SIRT1, and it significantly stimulated the protective effects of moderate Vit C [146]. The protective effect of Vit C against OS was related to the upregulation of SIRT1.

### 4.2. Activation of the Transcription Factor NRF2 by Polyphenols, Vitamins, and Selenium

The nuclear factor erythroid 2-related factor 2 (NRF2) responds to the OS by binding to the regions of genes that control the antioxidant response (ARE), and the NRF2/ARE induction pathway is of great interest due to its activation by phytochemical compounds [147]. NRF2 is tightly regulated at different levels by epigenetic modifications such as OS, inflammation, and different forms of stress [148], and has regulatory functions in mitochondrial biogenesis and cellular energy metabolism [149]. In other words, the NRF2 factor controls gene expression and the regulation of antioxidant and detoxifying enzymes [150] since it modulates the expression of more than 200 genes found in the promoter region of the antioxidant response (ARE) [151].

Under basal conditions, NRF2 is kept at a low cellular concentration by the activity of Keap1, its major regulator, keeping the expression of NRF2-regulated genes at low levels in order to preserve the correct redox homeostasis [152]. It is involved in the expression of the detoxification mechanism and cellular anti-apoptotic factors [153].

The NRF2 transcription factor can be activated by two mechanisms. The most common is based on the inhibition of its degradation in the cytosol, which is followed by its translocation to the nucleus, but it can also be activated by the effect of micromolar amounts of H_2_O_2_. The ex novo synthesis of NRF2 is triggered after exposure to low concentrations of H_2_O_2_, which precedes the translocation of NRF2 to the nucleus. Evidence for the ex novo synthesis of NRF2 is observed for low steady-state H_2_O_2_ concentrations, a condition that prevails in vivo [154].

#### 4.2.1. Transcription Factor NRF2 and Polyphenols

Polyphenols have the ability to activate NRF2 by inhibiting the Keap1-NRF2 protein–protein interaction [155]. Giovanni Scapagnini et al. presented data from their laboratory and others showing that curcumin strongly induces heme oxygenase-1 (HO-1) expression through activation of the NRF2/antioxidant response element (ARE) pathway [156]. The functions of this HO-1 enzyme are regulatory signaling, immunomodulatory, and cytoprotective [157], and the HO-1 gene has a consensus sequence in the ARE domain. The activation of HO-1 in neurons has a strongly protective effect against OS and cell death [158].

Hilla Erlank et al. proposed that polyphenols activate NRF2 in astrocytes through the production of H_2_O_2_, semiquinones, and quinones. Their study found that NRF2 translocation into the nucleus and NAD(P)H quinone oxidoreductase (NQO1) activity were significantly increased after treatment of astrocytes with tert-butylhydroquinone (tBHQ), resveratrol, or curcumin at 20–50 μM [159].

More recently, Joseph Kanner (also present in the aforementioned publication) supports the same thesis that polyphenols, by generating H_2_O_2_, affect redox signaling and activate the NRF2 axis to ensure cell adaptation and survival. This mechanism works via the following pathway: polyphenols generate H_2_O_2_ in the blood system in the endothelial cell membrane, thereby activating signaling factors. In other words, polyphenols act as reducing agents, but at the same time, they are pro-oxidants at the level of the blood system, so they act synergistically to maintain redox homeostasis in our organism and better health [80]. The produced H_2_O_2_ enters the cells via aquaporin, the protein channel generally associated with water transport, and triggers the activation of the NRF2 factor [160].

Currently, there is considerable interest in electrophilic drugs that act through NRF2 signaling and activation. A mechanism for NRF2 induction is via an electrophilic quinone [161], because they act as cofactors for electron transport in cellular respiration and, due to their semiquinone radicals, are capable of redox cycling and forming ROS [162]. In polyphenols, the reactions take place in equilibrium between the polyphenolic group, semiquinones, and quinones.

**Figure 11:**
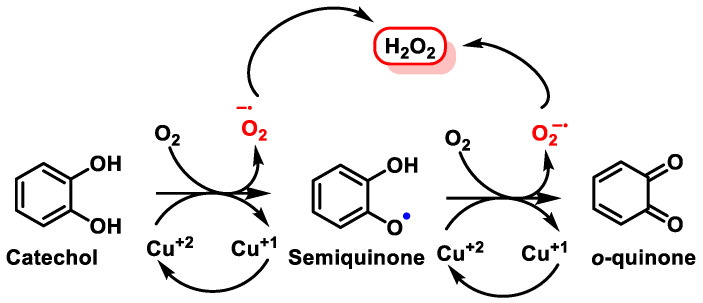
Mechanism of oxidation of the catechol group to o-quinones.

The transformation of the catechol group into o-quinones is a chemical reaction involving the oxidation of the hydroxyl group at the ortho position of the phenolic ring [163]. The enzymatic oxidation is catalyzed by a family of enzymes, such as cytochrome P450 (CYP), cyclo-oxygenase-2 (COX-2), peroxidase, tyrosinase (monophenol oxygenase), xanthine oxidase (XO), monoamine oxidase (MAO), and polyphenol oxidases (PPO), and is a reversible reaction using reducing agents such as H_2_ or H_2_S [164]. The reaction of o-quinones with the thiol group of Keap1 cysteines is an essential mechanism for the activation of the NRF2/ARE axis, regulating and ensuring the appropriateness of redox responses and oxidative signaling factors [165], (Figure 11). Under physiological conditions, catechol is oxidized in the presence of metals, or enzymatically in the presence of oxygen and metals, by an electron being transferred to molecular oxygen, resulting in the formation of ^•^O_2_^−^. In the presence of metals (e.g., copper, iron), ^•^O_2_^−^ is further reduced to H_2_O_2_. ROS can be harmful to cells and organisms if they are not removed.

**Figure 12:**
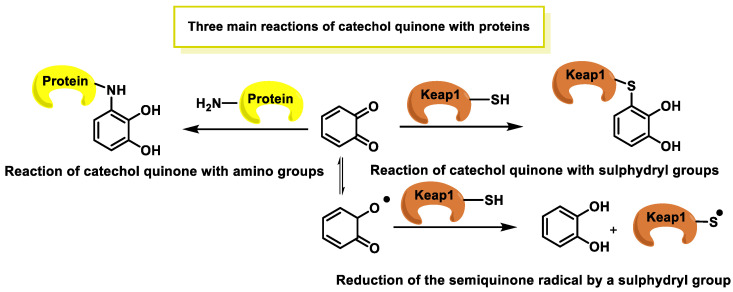
Interactions of catechols with proteins.

*O*-quinones, being Michael acceptor compounds and therefore electrophilic, can interact with certain cysteine residues in the Keap1 protein. This can alter the conformation of Keap1, inhibiting its ability to promote NRF2 degradation [166]. As a result, NRF2 accumulates in the cytoplasm and then translocates to the nucleus, where it can activate the ARE sequence. This in turn induces the expression of antioxidant and detoxifying genes, providing a defensive response against OS [167,168]. The o-quinones can covalently bind to nucleophiles such as cysteine, lysine, or histidine residues of proteins [169]. Three possible forms of interaction involve the reaction of o-quinone with sulfhydryl residues of cysteine or with amino groups of lysines, and a third possibility is the reduction of the semiquinone radical by a sulfhydryl group [170] (Figure 12).

**Figure 13:**
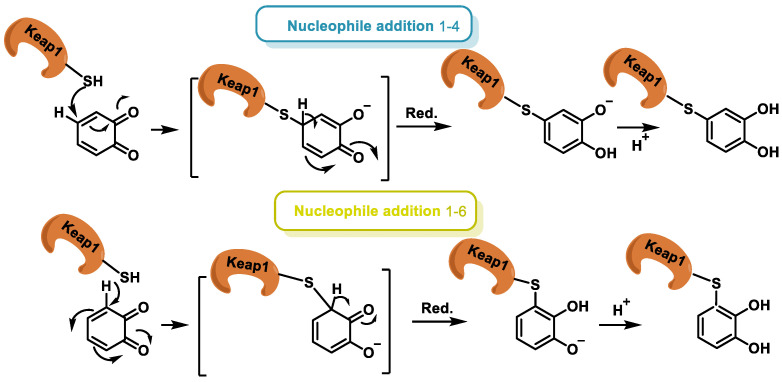
Possible mechanism of nucleophile 1–4 and 1–6 addition of sulfhydryl groups to o-quinone.

Addition of the nucleophile can take place via an attack on the carbon β (addition 1–4) or on the carbon at δ (addition 1–6). The possible mechanism is described below (Figure 13).

**Figure 14:**
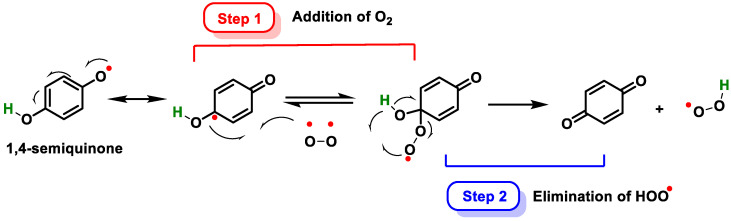
Addition of O_2_ to semiquinone to produce ^•^HO_2_. Underlies the pro-oxidant activity of hydroquinones.

Hydroquinone derivatives, when both hydroxyls are free, are easily transformed into p-quinones in the presence of oxygen or metal cations. The transition between the phenolic (reduced) and quinonic (oxidized) forms involves the participation of 2H^+^ and 2e^−^, and the redox potential of the process is strongly influenced by the pH of the medium and the nature of the solvent. The reaction is carried out in two steps: firstly, oxygen binds to the aromatic ring, and then the intramolecular H-atom transfer is concerted with cleavage of the hydroperoxyl moiety (Figure 14).

#### 4.2.2. Transcription Factor NRF2 and Vitamins

Chaweewan Sirakawin et al., 2023, used *Caenorhabditis elegans* to study the impacts of various bioactive compounds on lifespan. They demonstrated that Vit A extends lifespan and fat accumulation while increasing resistance to heat and OS. Vit A positively regulates NRF2 transcript levels in both *C. elegans* and human cells and mouse liver tissues. This study provides novel insights into the molecular mechanism of the anti-aging and antioxidant effects of Vit A, suggesting that this micronutrient could be used for the prevention and/or treatment of age-related disorders [171].

Li-Li Xu et al., 2020, studied whether OS plays a key role in the progression of severe acute pancreatitis (SAP). In vivo and in vitro results showed that Vit C treatment enhanced pancreatic acinar cell apoptosis, as evidenced by increased expression of Bcl-2, Bcl-XL, and MCL-1 and decreased expression of the apoptosis regulator Bax. The present study suggests that high doses of Vit C enhance pancreatic SAP injury through the NRF2 pathway to inhibit OS [172].

Mishra et al., 2019, investigated the protective role of Vit E in mitigating OS and restoring antioxidant potential in cardiac tissue under altered thyroid conditions. Based on the results obtained in the wet lab and in silico, they hypothesized that VIT-E inhibits LPx by reducing ROS and by increasing enzymatic antioxidant defense through binding of KEAP1, thus interfering with the NRF2-KEAP1 protein–protein interaction and leading to ARE activation. Administration of Vit E in a hyperthyroid state may be useful to mitigate cardiac damage in altered thyroid states in general, and to reduce the risk of hyperthyroidism-induced heart failure or stroke [173].

Vit D activates the NRF2-ARE antioxidant pathway. Nakai et al., 2013, explored whether maxacalcitol, an active vitamin D analogue, could also attenuate OS and prevent the progression of diabetic nephropathy. They concluded that maxacalcitol attenuates the progression of diabetic nephropathy by suppressing OS and enhancing the NRF2-Keap1 pathway in non-obese type 2 diabetes [174].

Cancer cells produce high levels of endogenous antioxidant enzymes that neutralize FR, seeking to compensate for intracellular ROS levels, and this mechanism facilitates the survival of neoplastic cells [175]. Positive regulation of the antioxidant NRF2-ARE axis provides protection to tumor cells against oxidative damage, and thereby stimulates tumor progression by increasing the aggressiveness and chemoresistance of tumor cells [176]. Mostafavi-Pour et al., 2017, investigated the role of Vit C and quercetin (Q) in the induction of NRF2-mediated OS in cancer cells, examining the antiproliferative effects of Vit C and Q. The results showed a significant decrease in NRF2 mRNA expression and protein levels after treatment of breast cancer cells with Vit C and Q: the nuclear/cytosolic NRF2 ratio was reduced 1.7-fold in MDA-MB 231 cells, 2-fold in MDA-MB 468 cells, 1.4-fold in MCF-7 cells, and 1.2-fold in A549 cells. The results of the current study suggest that Vit C and Q treatment may be developed as an adjuvant for cancer patients with NRF2 overexpression [177].

#### 4.2.3. Transcription Factor NRF2 and Selenium

Se deficiency, which compromises selenoprotein functions, and excess Se, which is toxic, have been associated with altered redox homeostasis and adverse health conditions. Se deficiency has been implicated in a wide range of chronic diseases, such as cancer, Alzheimer’s disease, and thyroid dysfunction. It can also affect the gut microbiota, potentially jeopardizing the human–microbiota symbiotic relationship and making the microbiota more susceptible to the development of diseases such as cancer, thyroid dysfunction, and cardiovascular disorders [123].

Interestingly, Se deficiency is associated with pro-longevity mechanisms because of reduced amino acid levels and altered nutrient signaling. The data show that the metabolic control associated with nutrient sensing coordinately responds to suppressed selenoprotein functions, resulting in a normal lifespan under Se deficiency. While Se deficiency can activate pathways linked to nutrient sensing and longevity, it also reduces the expression of selenoproteins, which play essential roles in pivotal physiological pathways [178].

Moderate Se deficiency can activate both the NRF2 and Wnt pathways. Under conditions of moderate Se deficiency, NRF2 target genes are induced. This induction is thought to compensate for the loss of selenoproteins and to help to maintain cellular redox balance [179]. The Wnt pathway, on the other hand, plays a crucial role in tissue and organ fibrosis. Studies have shown that moderate Se deficiency can lead to upregulation of the Wnt pathway. This upregulation is associated with changes in fibrosis marker proteins and components of the Wnt/β-Catenin signaling pathway [180]. Therefore, while Se deficiency can activate pathways linked to nutrient sensing and longevity, it also affects the expression of selenoproteins, which play essential roles in pivotal physiological pathways.

The Keap1/NRF2 system and the Wnt pathway have different roles and effects on the body, particularly in the context of Se deficiency. Under conditions of Se deficiency, the NRF2 pathway can be activated to help maintain cellular redox balance [152]. The Wnt pathway is often associated with risks. Activation of the Wnt pathway, as can occur in moderate Se deficiency, is associated with changes in fibrosis marker proteins and components of the Wnt/β-Catenin signaling pathway. This can lead to adverse health conditions, including carcinogenesis [181].

## 5. Transcriptional Regulation of Polyphenols, Vitamins, and Selenium

Polyphenols have been shown to modulate gene expression at several levels, including the following: (i) They can bind to transcription factors, and can either activate or repress transcription of specific genes [182]; (ii) they can affect the stability of mRNA, and can increase or decrease the production of specific proteins [183]; and (iii) they can also affect the translation of mRNA into proteins [184].

The modulation of gene expression by polyphenols can have a variety of biological effects. Polyphenols can increase the production of antioxidant enzymes and decrease the production of inflammatory proteins. In addition, polyphenols can modulate the production of genes involved in metabolism, cell signaling, and other processes [185].

### 5.1. Regulation of NF-κB

Proinflammatory transcription factors are members of the NF-κB and AP1 families, and their associated signaling cascades are activated by extracellular ligands and membrane-bound receptors, usually members of the Toll-like receptor superfamilies [186].

NF-κB participates in several physiological and pathological conditions, such as immune response, apoptosis, carcinogenesis, inflammatory processes, etc., and is a primary “fast-acting” transcription factor [187]. The canonical NF-kB pathway can be activated by OS and/or proinflammatory cytokines [188]. For NF-κB to be in its inactive state in the cytosol, it must be complexed with the inhibitory IκB protein. The function of the IKK kinase is to phosphorylate the IκB protein and contribute to the dissociation of the IκB/NF-κB complex, as well as the subsequent degradation of IκB by the proteosome [189]. When NF-κB has been released from the IκB protein, it is activated and translocates to the nucleus, where it binds to specific DNA sequences called response elements (RE) and expresses the production of proteins and enzymes that cause changes in the physiological metabolism of the cell [190], such as in the inflammatory, immune, and survival responses and the cell proliferation response [191]. In neoplastic cells, activation of NF-κB provides the ability to survive by upregulating anti-apoptotic genes, including several members of the BCL-2 family [192], and increases resistance to chemotherapy by controlling the expression of multidrug resistance gene 1 (mdr1) [193].

Several studies have explored the ability of polyphenols to regulate NF-κB signaling and have revealed that they exert repressing effects on NF-κB activation through a diversity of mechanisms [194]:

- They can inhibit the activity of IKK, thereby preventing the phosphorylation and subsequent degradation of IκB proteins. This action blocks the translocation of NF-κB to the nucleus, preventing it from activating gene expression [195].
- Indirectly inhibiting NF-κB activation due to its antioxidant properties [196].
- Can influence the composition of NF-κB subunits, thereby altering the activity of the NF-κB complex. The p65 subunit, also known as RelA, is a key component of the NF-κB complex and plays a crucial role in the transcriptional activity of NF-κB. This inhibition can prevent the translocation of NF-κB into the nucleus and the transcription of pro-inflammatory cytokines [197].
- Can disrupt upstream signaling pathways of NF-κB activation, as they can interfere with Toll-like receptors (TLRs) [198] or cytokine receptors [199], which are crucial for the initiation of NF-κB signaling cascades. By doing so, polyphenols can inhibit the activation of NF-κB, thereby potentially reducing the expression of NF-κB-dependent genes, many of which are involved in inflammatory responses.

Tumor necrosis factor alpha (TNF-α) activates NF-kB through well-defined kinase pathways. Intracellular Vit C inhibits TNF-α-induced NF-kB activation in human cell lines (HeLa, monocytic U937, myeloid leukemia HL-60 and breast MCF7) and primary endothelial cells (HUVEC) in a dose-dependent manner. The data point to a mechanism of suppression of NF-kB activation by vitamin C through inhibition of TNF-α-induced activation of the p38 MAP kinase-independent kinases NIK and IKKβ. These results suggest that intracellular vitamin C may influence inflammatory, neoplastic, and apoptotic processes by inhibiting NF-kB activation [200]. Liv Austenaa et al., 2004, obtained a similar result with Vit A [201]. The effect of vitamin E on NF-kB activation has been examined in many studies, using both in vivo and in vitro models. Most of these studies have found that vitamin E inhibits NF-kB activation, with the greatest inhibition observed with the succinate form. This effect may be due to a reduction in OS [202].

Carole Kretz-Remy et al., 2001, studied the role of Se in NF-κB activation, analyzing in human T47D cells the overexpression of the seleno-dependent detoxifiant enzyme glutathione peroxidase. Following exposure to H_2_O_2_, these cells showed a seleno-dependent decreased accumulation of intracellular ROS and NF-κB activation. This phenomenon was correlated with an inhibition of the nuclear translocation of NF-κB (p50 subunit) and with an absence of IκBα degradation. They also reported that the half-life of IκBα in untreated cells was increased twofold by the overexpression of active glutathione peroxidase. Their results suggest that Se can modulate glutathione peroxidase activity, can inhibit NF-κB activation, and can increase the normal half-life of IκBα [203].

### 5.2. Regulation of AP-1

The Activator Protein-1 (AP-1) is a dimeric transcription factor. It is involved in various cellular events, including differentiation, proliferation, survival, and apoptosis. AP-1 is also a critical regulator of nuclear gene expression during T-cell activation and is one of the downstream targets of the MAPK signaling cascade [204]. It is activated by OS, inflammation, viral or bacterial infections, and DNA damage [205]. The dysregulation of AP-1 can lead to a variety of diseases, such as cancer, inflammation, and neurodegenerative disorders [206]. Polyphenols can inhibit AP-1 activity by (i) blocking the binding of AP-1 to DNA, (ii) inactivating the AP-1 proteins, and (iii) interfering with the signal transduction pathways that activate AP-1 [207]. The inhibition of AP-1 activity can decrease cell proliferation; reduce inflammation; and increase cell differentiation, apoptosis, and antioxidant activity [208].

S. A. Mattmiller et al., 2013, showed that many of the health benefits of Se are thought to be due to the antioxidant and redox-regulating properties of certain selenoproteins. Optimal Se intake can mitigate dysfunctional inflammatory responses, in part through the regulation of eicosanoid metabolism [209].

### 5.3. Regulation of STAT3

The STAT (Signal Transducer and Activator of Transcription) family of proteins act primarily as signal transducers and activators of transcription, participating in processes of proliferation, immunity, apoptosis, and cell differentiation [210]. Once activated by phosphorylation, they move into the cell nucleus to carry out gene transcription of some genes. In the presence of cytokines and growth factors, STAT3 is phosphorylated by receptor-associated tyrosine kinases, and this phosphorylation allows STAT3 to form homo- or heterodimers that translocate to the cell nucleus, where they act as activators of transcription [210]. STAT3 plays an important role in a multitude of cellular processes, such as cell proliferation and apoptosis. The STAT3 protein, for example, may contribute to uncontrolled cell proliferation, which can lead to tumor formation [211]. STAT3 has emerged as a promising target for cancer drug development [212]. Polyphenols have been shown to inhibit STAT3 activity, and this might contribute to beneficial health effects, especially in the prevention of cardiovascular disease and type 2 diabetes [213]. However, the exact relationship between polyphenols and STAT3, as well as the specific mechanisms of inhibition, are areas of active research.

Ming Zhang et al., 2013, studied the phosphorylation activity of mitochondrial signal transducer STAT3 in the myocardium of rats with Se deficiency and its association with myocardial injury. Se deficiency was shown to down-regulate the activity of mitochondrial STAT3 in rat hearts, thus contributing to cardiac mitochondrial injury and the progression of heart failure [214].

### 5.4. Regulation of BACH1

BACH1 (BTB Domain And CNC Homolog 1) is a protein-coding gene [215]. It encodes a transcription factor that belongs to the cap’n’collar type of basic region leucine zipper factor family (CNC-bZip). The encoded protein contains broad complex, tramtrack, bric-a-brac/poxvirus, and zinc finger (BTB/POZ) domains, which is atypical of CNC-bZip family members [215]. These BTB/POZ domains facilitate protein–protein interactions and the formation of homo- and/or hetero-oligomers. When BACH1 forms a heterodimer with MafK, it functions as a repressor of MAF recognition element (MARE), and transcription is repressed [216]. BACH1 plays important roles in coordinating transcription activation and repression by MAFK. It also plays crucial roles in OS, the cell cycle, hematopoiesis, and immunity. BACH1 has been associated with diseases such as breast cancer [217]. It has been shown to function as an inducer of metastatic genes in breast cancer, including CXCR4 and MMP1 [218]. BACH1 is involved in various physiological processes and pathogenesis related to inflammation, oxidative stress damage, autoimmunity disorders, and cancer angiogenesis, among others [219].

BACH1 and NRF2 are both involved in the regulation of the antioxidant response in cells. They interact with each other in a competitive manner to regulate antioxidant response element (ARE)-mediated gene expression. In cells that are naïve to OS, BACH1 binds to ARE-like enhancers and antagonizes NRF2 binding until it becomes inactivated by pro-oxidants [220]. This means that BACH1 can prevent NRF2 from activating the transcription of certain genes under normal conditions [221].

When cells are exposed to OS, BACH1 becomes inactivated, which allows NRF2 to bind to the ARE and induce the expression of protective antioxidant genes [222]. For example, the induction of the heme oxygenase-1 (HMOX1) gene, which is elicited by arsenite-mediated OS, follows the inactivation of BACH1 and precedes the activation of NRF2. In summary, the relationship between BACH1 and NRF2 is a dynamic one, with BACH1 acting as a repressor and NRF2 as an activator of ARE-mediated gene expression. The balance between these two factors can influence the cellular response to OS [223].

BACH1 and o-quinones are both involved in the regulation of OS responses in cells. The electrophilic character of o-quinones is essential for the suppression of BACH1. When cells are exposed to OS, such as that caused by o-quinones, BACH1 becomes inactivated. This allows NRF2 to bind to the ARE and induce the expression of protective antioxidant genes [218]. For example, the induction of the heme oxygenase-1 (HMOX1) gene, which is elicited by OS, follows the inactivation of BACH1 and precedes the activation of NRF2 [224]. In summary, the relationship between BACH1 and o-quinones is a dynamic one, with BACH1 acting as a repressor and o-quinones contributing to its inactivation under OS conditions. This balance influences the cellular response to OS [225].

Ting Wang et al., 2023, have demonstrated that BACH1 controls the transcription of a broad range of angiogenesis genes and is stabilized by lowering ROS levels. Xenograft tumors (generated through the implantation of human tumors in mice) increased substantially following the administration of vitamins C and E and N-acetylcysteine in a BACH1-dependent fashion under normoxia. Moreover, angiogenesis gene expression increased in endogenous BACH1-overexpressing cells and decreased in BACH1-knockout cells in the absence of antioxidants [226].

## 6. Polyphenol-Mediated Enzyme Regulation

Polyphenols are involved in enzyme regulation, with a wide range of biological activities, and can interact with proteins through hydrophobic interactions, hydrogen bonding, and electrostatic interactions [227]. These interactions can influence the function of enzymes, potentially altering their activity.

### 6.1. NADPH Oxidase

**Figure 15:**
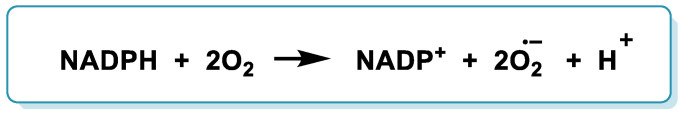
Reaction for the formation of ^•^O_2_^−^ from NADPH.

Elevated levels of ROS are constitutive in cancer. They are an important hallmark derived from increased production in mitochondria and by the NADPH oxidase (NOX, nicotinamide adenine dinucleotide phosphate oxidase) family of enzymes [228]. NOX is a membrane-bound enzyme complex that faces the extracellular space, and it can be found in the plasma membrane as well as in the membranes of phagosomes used by neutrophil white blood cells to engulf microorganisms. NADPH oxidase catalyzes the production of an ^•^O_2_^−^ by transferring one electron to O_2_ from NADPH [14]. The overall reaction for the formation of ^•^O_2_^−^ from NADPH is as follows (Figure 15):

If NOX expression is not properly regulated, NOX-associated ROS can promote OS, aberrant signaling, and genomic instability [229]. NOX isoforms are already known to be overexpressed in multiple malignancies, making them potential therapeutic targets in cancer. If NOX expression is not properly regulated, NOX-associated ROS can promote OS, aberrant signaling, and genomic instability [229]. NOX isoforms are already known to be overexpressed in multiple malignancies, making them potential therapeutic targets in cancer [230].

Several studies have investigated the potential of polyphenols to inhibit NOX activity and reduce ROS production, and it has been observed that these natural compounds prevent NOX expression [90]. Several compounds that have been studied are resveratrol [231], quercetin [232], EGCG [233], and curcumin [234]. The possible mechanism by which polyphenols inhibit NOX are by blocking the assembly of the NOX complex, necessary for the enzyme activity [235], and acting in the NOX electron transport chain [236].

### 6.2. Cyclooxygenase 2

COX-2, also known as cyclooxygenase 2 or prostaglandin-endoperoxide synthase 2, is an enzyme that plays a key role in the biosynthesis of prostanoids, which include prostaglandins, prostacyclins, and thromboxanes. This enzyme is inducible, meaning that it is not normally detected in most tissues, but its production can increase in response to certain stimuli, such as inflammation [237]. However, in some structures such as the ovary, prostate, kidney, and central nervous system, COX-2 may have a structural character. It is important to mention that, although COX-2 has traditionally been seen as an enzyme that is expressed only under pathological conditions, it has detrimental effects on the pathophysiology of diseases such as Alzheimer’s disease [238]. In relation to COX-2, polyphenols may have several effects: (i) Some studies have suggested that polyphenols may inhibit COX-2 activity [239], and (ii) wine polyphenols have been shown to exert an antineoplastic effect on the androgen-resistant PC-3 cell line through inhibition of NF-κβ-mediated transcriptional activity of the COX-2 promoter. This could explain, at least in part, the induction of apoptosis in vitro by these substances in castration-resistant prostate cancer [240].

### 6.3. Lysyl Oxidase

Lysyl oxidase (LOX) plays an important role in extracellular matrix (ECM) stabilization and may be related to endothelial dysfunction induced by atherosclerotic risk factors [241]. Inhibition of LOX may impair endothelial barrier function. In addition, it has been proposed that it has roles in atherogenesis and endothelial dysfunction, ocular disorders, fibrosis, iatrogenic diseases, bone regeneration, and increased risk of cardiovascular diseases, among others [242]. LOX catalyzes the oxidative deamination of lysine and hydroxylysine residues in collagen and elastin, two major structural proteins found in the ECM. This reaction produces aldehydes, which then form covalent cross-links between collagen and elastin molecules, strengthening the ECM and providing resistance to mechanical forces [243]. Polyphenols can influence the activity of LOX in different ways. Some polyphenols, such as chlorogenic acid, gallic acid, and caffeic acid, have been shown to have amine oxidase-like activity, which means that they can mimic the action of LOX and participate in collagen cross-linking. This suggests that polyphenols may contribute to the strengthening of the ECM and the maintenance of tissue integrity [244].

### 6.4. Lipoxygenase

Lipoxygenases (LOs) are a family of enzymes that catalyze the addition of oxygen to polyunsaturated fatty acids (PUFAs), specifically those containing a 1,4-pentadiene structure [245]. This reaction results in the formation of hydroperoxides, which can then be further metabolized into a variety of bioactive molecules, including leukotrienes, hydroxyeicosatetraenoic acids (HETEs), and fatty acid epoxides [246]. Los are found in a wide range of organisms, including plants, animals, and fungi, and dysregulation of LO activity can have significant implications in various diseases [247]. Polyphenols can affect the activity of enzymes such as LO, modulating the inflammatory process [72]. This is thought to be one of the mechanisms by which polyphenols can help to reduce inflammation and protect against chronic diseases [248].

### 6.5. Xanthine Oxidase

**Figure 16:**
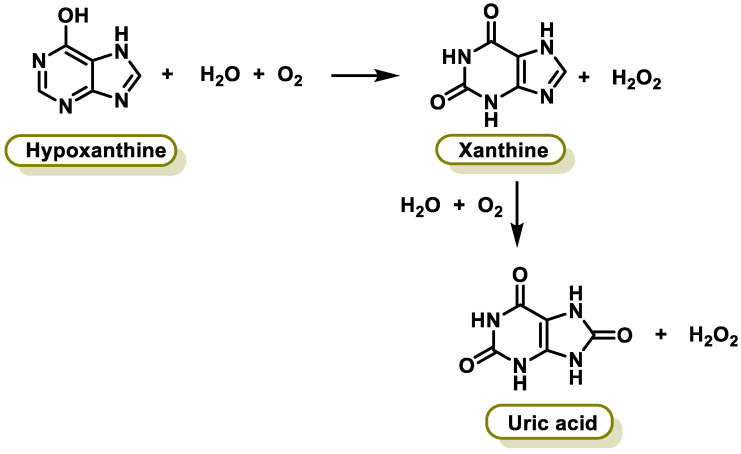
Chemical reactions catalyzed by xanthine oxidase.

Xanthine oxidase (XO) is a form of xanthine oxidoreductase, a type of enzyme that generates ROS. These enzymes catalyze the oxidation of hypoxanthine to xanthine and can further catalyze the oxidation of xanthine to uric acid [249]. They play an important role in the catabolism of purines in some species, including humans. The following chemical reactions are catalyzed by XO (Figure 16):

Inhibition of xanthine oxidase reduces the production of uric acid, and several medications that inhibit xanthine oxidase are indicated for treatment of hyperuricemia and related medical conditions. Polyphenols, particularly flavonoids, are known to have antioxidant properties and can act as potent inhibitors of XO activity [250]. XO is the main contributor of FR during exercise, but it is also involved in the pathogenesis of several diseases such as vascular disorders, cancer, and gout [251]. Several studies have indicated that the capacity of flavanols and flavones to inhibit the active site is largely dependent on hydrogen bonds between the polyphenol ligand hydroxyl groups and the catalytic residues of the binding site [252].

### 6.6. α-Synuclein

α-synuclein (αS) is a neuronal protein that is abundantly expressed in the brain, specifically in presynaptic nerve endings, constituting more than 1% of the total protein in the cytosol of brain cells. It is the major component of Lewy bodies in both sporadic and inherited forms of Parkinson’s disease and in Lewy body dementia. αS is a key protein in the pathology of Parkinson’s disease (PD) [253], characterized by the loss of dopaminergic neuronal cells in the substantia nigra pars compacta and the abnormal accumulation and aggregation of αS in the form of Lewy bodies and Lewy neurites. αS is the main component of Lewy bodies and is a pathogenic feature of all synucleinopathies, including Parkinson’s disease (PD), dementia with Lewy bodies (DLB), and multiple system atrophy (MSA). All of these diseases are determined by the deposition of αS aggregates, but can be separated into distinct pathological phenotypes and diagnostic criteria [254]. Kenjiro Ono et al., 2020, studied the impact of the polyphenolic acids 3-hydroxybenzoic acid (3-HBA), 3,4-dihydroxybenzoic acid (3,4-diHBA), and 3-hydroxyphenylacetic acid (3-HPPA) (derived from gut microbiota-based metabolism of dietary polyphenols) on the brain, and demonstrated their ability to inhibit αS oligomerization and mediate aggregate αS-induced neurotoxicity in vitro [255].

### 6.7. Receptor Tyrosine Kinases

Receptor tyrosine kinases (RTKs) are transmembrane proteins that act as signal transducers. They regulate essential cellular processes such as proliferation, apoptosis, differentiation, and metabolism. RTKs play an important role in cancer progression and are activated in response to environmental signals by initiating appropriate signaling cascades in tumor cells [256]. Alteration of RTKs occurs in a broad spectrum of cancers, emphasizing their crucial role in cancer progression and as a suitable therapeutic target [256]. It has been demonstrated that EGCG, a type of polyphenol, can lower levels of EGFR, a type of RTK, by both inhibiting transcription of the encoding gene and inducing internalization followed by degradation [257]. Another study identified tyrosine kinase inhibitors from *Panax bipinnatifidus* and *Panax pseudoginseng*, which are rich in polyphenols [258].

### 6.8. Histone Deacetylases

Histone deacetylases (HDACs) are a class of enzymes that remove acetyl groups from lysine residues on histone proteins. These modifications play a crucial role in regulating gene expression by altering the chromatin structure. The removal of acetyl groups by HDACs leads to the condensation of chromatin, making the DNA more tightly wound around histone proteins [259]. This compact chromatin structure hinders the access of transcription factors and RNA polymerases, consequently repressing gene expression. Conversely, histone acetylation by histone acetyltransferases (HATs) loosens chromatin, facilitating gene transcription [260]. HDACs are involved in a wide range of biological processes, including cell growth, differentiation, apoptosis, and metabolism. Dysregulation of HDAC activity has been implicated in several diseases, such as cancer, neurodegenerative disorders, and metabolic disorders [261]. HDAC inhibitors are a class of drugs that selectively inhibit HDAC activity. These inhibitors are being investigated as potential therapeutic agents for various diseases, including cancer, Alzheimer’s disease, and type 2 diabetes [262]. By inhibiting HDAC activity, these drugs aim to reverse the repressive effects of HDACs on gene expression and restore normal cellular function. Some flavonoids have been reported to act as HDAC inhibitors [263]. Choi et al., 2016, reported that piceatannol (a resveratrol metabolite found in red wine) affected HDAC expression in a mouse model and concluded that it may be a valuable therapeutic agent in renal fibrosis by decreasing HDAC4 and HDAC5 protein expression [264].

### 6.9. α-Amylase and α-Glucosidase

α-amylase and α-glucosidase are two important enzymes involved in carbohydrate digestion. They play crucial roles in breaking down starch, the main carbohydrate found in grains, legumes, and vegetables, into smaller sugar molecules that can be absorbed by the body. α-amylase is found in both salivary glands and the pancreas. It acts on starch by breaking down α-1,4-glycosidic bonds, which are the bonds that connect glucose units together. This process produces smaller chains of glucose molecules called dextrins and maltose. α-glucosidase is also found in the pancreas and small intestine. It further breaks down dextrins and maltose into glucose, the simplest form of sugar. This allows glucose to enter the bloodstream and be used for energy [265].

Inhibition of these two enzymes can be a useful strategy for controlling blood sugar levels in people with diabetes. This is because it can slow down the digestion of carbohydrates, which helps to prevent rapid spikes in blood sugar after eating. There are a number of different ways to inhibit α-amylase and α-glucosidase, including dietary strategies, natural inhibitors, and pharmaceutical agents (these agents are typically used to treat type 2 diabetes) [266].

There are several reports indicating the anti-diabetic capabilities of polyphenols through the inhibition of carbohydrate-hydrolyzing enzymes [267]. Flavonoids are explored as inhibitors of α-amylase, whereas polyphenols are thought to regulate starch digestibility [268]. According to Lo Piparo et al., 2008, the efficacy of inhibition is usually correlated with the amount of OH on the B-ring of the flavonoid [269].

## 7. Is Bioavailability an Important Issue in the Functionality of Antioxidants?

Plant antioxidants encompass various compounds, including vitamins, polyphenols, and tocopherols. These natural antioxidants have gained significant interest due to their potential to prevent CVD, cancer, neurodegenerative disorders, diabetes, and other diseases [270].

Bioavailability refers to the amount of a nutrient that can be absorbed and utilized by the body. It involves the rate and speed at which a drug or nutrient reaches its therapeutic target and the target tissue through channels, transporters, or receptors [271].

Vit C is water-soluble, and its bioavailability is dependent on the dose. In humans, transport saturation occurs at doses of 200–400 mg per day, with approximately 70% absorption of a 500 mg dose. However, about 50% of the absorbed dose is not metabolized and is excreted in the urine [272]. Vit C is essential for collagen synthesis, L-carnitine biosynthesis, and the production of certain neurotransmitters. As an antioxidant, it can regenerate other antioxidants, such as α-tocopherol [273]. Factors influencing Vit C bioavailability include glucose ingestion, which decreases bioavailability; synergistic effects with polyphenols that increase bioavailability by up to 35%; increased iron bioavailability, but not zinc bioavailability; oxidation processes affecting bioavailability in the presence of oxygen; and lifestyle factors like smoking, physical inactivity, and obesity, which can decrease Vit C bioavailability [274].

Vit E is a fat-soluble vitamin with a high dietary bioavailability of about 50–80%. It follows the general absorption pathway of fats and can be absorbed even without dietary fats. However, simultaneous consumption of fats can enhance its absorption in the small intestine [275]. Vit E is mainly absorbed from vegetable oils and is associated with lipids like triacylglycerols, cholesterol, and phospholipids. It is minimally broken down in the stomach, and partial release by the action of pepsin has been suggested. Further release occurs through the action of digestive enzymes, such as pancreatic lipase, in the duodenum [276]. In the stomach, food is mixed with gastric secretions and exposed to the acidity and enzymes of the stomach. Tocopherol is not appreciably broken down here, but it is thought to be partially released by the action of pepsin [277]. The amount of Vit E already present in vegetable oils or nuts is transferred to dietary fat, and this process depends on the characteristics of the food and the amount and type of dietary fat. Digestive enzymes in the duodenum, proteases, amylases, and lipases continue to release Vit E from the food matrix [278].

Polyphenols, from a chemical perspective, act as antioxidants with greater capacity compared to the previously mentioned antioxidants. Therefore, their absorption and bioavailability are crucial. However, the bioavailability of polyphenols is often low due to interactions with absorption processes mediated by the liver, intestine, and microbiota. Flavonoid aglycones, like quercetin, are generally poorly water-soluble, further limiting their bioavailability. Additionally, the biological activities of phenolic compounds can be influenced by their metabolites [279,280,281].

Pharmacokinetic studies have provided data on the bioavailability of different polyphenols, with the following order: phenolic acids > isoflavones > flavonols > catechins > flavanones > pro-anthocyanidins > anthocyanins [282].

While several epidemiological and clinical studies have explored the benefits of polyphenol consumption, limitations such as small sample sizes, lack of controls, varied methodologies, and heterogeneous data correlations have been observed. However, well-designed studies like PREDIMED, which focused on the Mediterranean diet characterized by high polyphenol intake, have shown reduced cardiovascular risk and improved cognitive function in the elderly [283,284]. Hydroxytyrosol, found in olive oil, and its derivatives, such as oleuropein, may contribute to the beneficial effects of the Mediterranean diet. Currently, hydroxytyrosol is the only polyphenol with an approved health claim related to its phenolic compound content [285].

In addition to considering the link between bioavailability and health effects, the average daily intake of polyphenols is also important. A systematic review estimated an average daily polyphenol intake of 0.9 g/day in the general population (including adolescents, adults, and the elderly). The main dietary sources of polyphenols were coffee, tea, red wine, fruits, and vegetables. This intake was associated with a reduction in CVD and type 2 diabetes mellitus (T2DM) [286].

## 8. Can Antioxidants Act as Pro-Oxidants?

Pro-oxidants are substances that can increase OS in the body, which can result in damage to cellular components and contribute to the development of various diseases. The role of exogenous antioxidants in preventing or delaying oxidative damage has become increasingly controversial [287]. It is important to note that taking antioxidants as supplements may not always be effective and can even be potentially dangerous. High doses of Vit E are associated with an increased risk of hemorrhagic stroke [288] and prostate cancer [289].

OS itself has a dual character in the body. While excessive OS is harmful and can cause damage, it is also a natural part of cellular signaling processes. Some ROS, which are produced during OS, act as signaling molecules in cellular pathways. Therefore, reducing OS through the use of antioxidant supplements may not always be beneficial in cases where ROS play important roles in cellular signaling [290,291]. Pro-oxidant substances can act through two main pathways:

- Increasing the formation of ROS: Certain substances can increase the production of ROS, which, in turn, can act as pro-oxidants themselves. This can lead to a cycle of oxidative damage and further increases in OS.
- Hindering the action of antioxidant enzymes and pathways: Pro-oxidants can interfere with the activity of antioxidant enzymes and pathways in the body, reducing their effectiveness in terms of neutralizing ROS and protecting against oxidative damage.

Many pro-oxidant substances specifically target and damage mitochondria, the cellular organelles responsible for energy production. Mitochondrial damage can disrupt the production of sufficient energy for vital cellular functions, leading to cellular dysfunction, tissue damage, accelerated aging, and the development of degenerative diseases [292].

### 8.1. Pro-Oxidant Function of Vitamins

**Figure 17:**
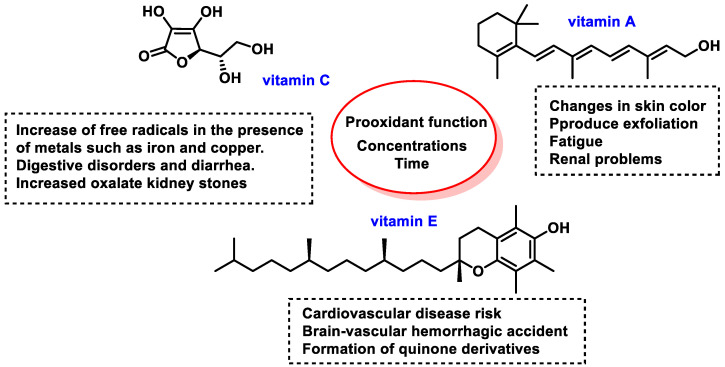
High concentrations of vitamins A, C, and E may have undesirable pro-oxidant effects.

Some of the pro-oxidant functions of vitamins A, C, and E are shown in Figure 17.

High concentrations of certain vitamins, such as vitamins A, C, and E, can have undesirable pro-oxidant effects, potentially increasing the risk of a heart attack [293,294]. It is important to note that, while these vitamins are generally beneficial and necessary for the body, excessive intake can lead to unintended consequences.

The intake of Vit C is particularly controversial. Linus Pauling, a renowned scientist, recommended a high daily dose of 1000 mg [295]. However, even at low concentrations, Vit C can exhibit a pro-oxidant effect in the presence of transition metals like iron. Ascorbic acid, the active form of Vit C, can increase FR production when it interacts with metals such as iron and copper, as it has the ability to reduce Fe^3+^ and Cu^2+^ to Fe^2+^ and Cu^+^ [296]. While Vit C is a direct antioxidant, its pro-oxidant potential in specific conditions needs to be considered.

Adverse effects of high doses of Vit C can include indigestion, diarrhea, and an increased risk of oxalate kidney stone formation. It is worth noting that Vit C is one of the most widely used dietary supplements in the United States [297].

Vit E, when converted to quinone derivatives, can be toxic to cells and produce oxygen radicals. While Vit E is generally well tolerated by the body, high doses may interfere with the body’s utilization of other fat-soluble vitamins. Conflicting data regarding high-dose Vit E supplementation and its adverse effects have been published in recent years, emphasizing the need for further investigation [298]. Excessive doses of Vit A or retinol can surpass the liver’s ability to store the vitamin, leading to intoxication. This can result in adverse effects such as changes in skin color and peeling of the skin. A balanced and varied diet that includes a wide range of nutrients is generally the best approach to obtaining vitamins and minerals in appropriate amounts.

### 8.2. Pro-Oxidant Function of Polyphenols

A diet rich in flavonoids, a class of plant metabolites, has been associated with potential health benefits. However, it is important to note that a high intake of flavonoids may also have harmful effects due to their diverse pharmacological properties. Some flavonoids can act as pro-oxidants, which means that they have the potential to generate ROS and exhibit mutagenic and genotoxic effects in certain experimental systems [299]. Flavonoids can exert their pro-oxidant effects through various mechanisms, including the transient reduction of Cu^2+^ to Cu^+^, the formation of ROS, and the potential disruption of components of the antioxidant defense system in the nucleus, such as glutathione and glutathione-S-transferase [300]. These metabolites can reduce Fe^3+^ and Cu^2+^ and undergo auto-oxidation due to their structural properties.

It is important to note that flavonoids can exhibit both antioxidant and pro-oxidant properties, and their behavior may be influenced by factors such as test conditions, effective concentration at the site of ROS formation, stability of the flavonoid radical formed during hydrogen atom donation, lipophilicity for membrane absorption, and pH of the medium. Doses of flavonoids should not exceed those typically absorbed through a typical vegetarian diet to avoid excessive ROS formation and subsequent DNA damage [301].

**Figure 18:**
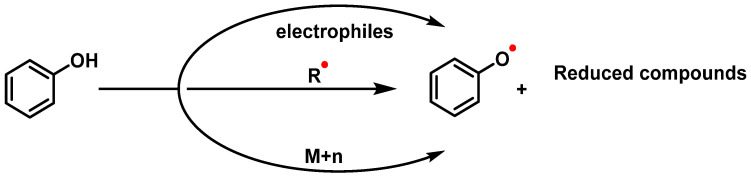
Dissociation of a phenolic OH group to phenoxyl radical.

The stability and redox capacity of the radical formed from the original flavonoid is the decisive factor in determining whether it exhibits antioxidant or pro-oxidant characteristics [302]. Pro-oxidant effects of flavonoids generally occur at high doses [303]. In the presence of oxygen and transition metals like iron and copper, dietary phenolic compounds, including flavonoids, can act as pro-oxidants by catalyzing the redox cycle of phenolic compounds. This can lead to the generation of ROS and phenoxyl radicals, which can cause damage to DNA, lipids, and other biological molecules [80]. Polyphenols are good reducing agents, but, according to this author’s thesis, they also have the potential to act as pro-oxidants. Both effects are related to the aromatic structure, its resonant electronic configuration, and the ability of the OH to donate a reducing group H^+^ to a free radical, which increases its redox activity. Figure 13 characterizes the ways in which the phenoxyl group is transformed into a radical. In polyphenols, the phenolic OH has a lower dissociation enthalpy than the OH of an aliphatic alcohol (such as ethanol or propanol), so that dissociation can occur in a slightly basic medium in the presence of a radical ^•^R or a bivalent metal cation (Figure 18).

The prooxidant effects of phenolic compounds are correlated with the one-electron redox potential of the phenoxyl radicals. There is a direct relationship between the prooxidant capacity and the high stability of the phenoxyl radicals formed, in this case because the odd electron is delocalized throughout the benzene ring [304].

In practice, the phenolic OH groups in polyphenols act as reducing elements, but at the same time, when the phenoxyl radical is formed, they function as pro-oxidants, which supports from a theoretical point of view the fact that these compounds are activators of the Nrf2 factor, as they interact with the Keap1 protein. This interaction allows the transcription factor to translocate to the cell nucleus and allows the genes present in the ARE domain to be expressed.

### 8.3. Pro-Oxidant Function of Selenium

In humans, according to World Health Organization (WHO) standards, the recommended dose of Se for adults is 55 μg/day, while the maximum tolerable adult intake without side effects is set at 400 μg/day [273]. Se-rich foods are a significant measure to avoid Se deficiency, but supplemental intake beyond the amounts needed for full expression of selenoproteins may be a health risk and is therefore not recommended [305]. In Venezuela, the fruit of the species *Lecythis ollaria*, also known as paradise nuts, is known to accumulate high amounts of Se. Excessive consumption of these seeds can lead to Se poisoning, which manifests as nausea, vomiting, and diarrhea, followed by hair loss and damage or loss of nails. While Se is an essential element for humans, it can be toxic at high levels [306].

Se has dual roles as both an antioxidant and a pro-oxidant. At low concentrations, it acts as an antioxidant, inhibiting lipid peroxidation, because Se is involved in the antioxidant defense systems. It is a key component of selenoproteins such as thioredoxin reductase (TR) and the glutathione peroxidase family (GPx), which have reactive oxygen species (ROS) scavenging activity [307].

On the other hand, Se can also act as a pro-oxidant, especially at higher concentrations. It is a pro-oxidant, enhancing the accumulation of lipid peroxidation products. In this role, Se can generate ROS, leading to oxidative stress. This pro-oxidant effect of Se is associated with its various oxidation states (Se^+6^, Se^+4^, Se^−2^) and is particularly evident with Se nanoparticles, which have reduced toxicity compared to elemental Se [308]. The balance between the antioxidant and pro-oxidant effects is crucial, as it can influence various biological processes and health outcomes. For example, in ryegrass, low concentrations of Se act as an antioxidant, inhibiting lipid peroxidation, whereas at higher concentrations, it is a pro-oxidant, enhancing the accumulation of lipid peroxidation products. This dual role of Se highlights its complex interactions within biological systems [309].

Se-containing molecules are a potential innovative therapeutic option against cancer and have been extensively investigated in recent years in cancer therapy in relation to tumor development and dissemination, drug delivery, multidrug resistance (MDR), and immune-related (anti)carcinogenic effects [310]. Historically, Se was considered both a potential toxin and a protective element. Contemporary research has revealed that Se can have both beneficial and harmful effects on organisms, including the nervous system and the heart [311]. Several human studies have demonstrated that supplementing standard-of-care chemotherapies (such as cisplatin, doxorubicin, cyclophosphamide, and busulfan) with Se (in the form of sodium selenite or organic selenium) reduces toxicity without compromising therapeutic efficacy [310]. Se-containing molecules have been investigated in various contexts: (i) tumor development and dissemination, (ii) drug delivery; (iii) multidrug resistance (MDR); and (iv) immune system modulation [312,313].

Selenites are known to undergo oxidation and reduction reactions, leading to the generation of their divalent cations (Se^2+^), endowed with oxidant properties [110]. Particular attention has been paid to the potential usefulness of Se-containing compounds in acting as anticancer and chemopreventive agents, inducing antioxidant and pro-oxidant effects at low and high doses, respectively [314]. Se-containing molecules can affect gene expression, cell signaling pathways, DNA repair/damage, and angiogenesis and metastasis through the formation of ROS and the oxidation of protein thiol groups [315]. Selenium nanoparticles (SeNPs) are emerging as a novel therapeutic platform, with reduced toxicity and the ability to enhance the biological properties of Se-based compounds [316]. SeNPs are useful tools in current biomedical research, with exceptional benefits as potential therapeutics, including enhanced bioavailability and improved targeting and efficacy against oxidative stress and inflammation-mediated disorders [317].

## 9. Conclusions

In this review, we have focused on three aspects: the reactivity of FRs, their cellular effects, and the potential use of antioxidants as scavengers of FRs and their role in cellular metabolism. In cells, FRs can modify the molecular structures of proteins and lipids, alter enzyme homeostasis, and cause structural damage to nuclear and mitochondrial DNA and various cellular organelles, contributing to the development of functional or gene expression abnormalities.

The modern lifestyle induces increased susceptibility to the effects of ROS. More than 100 ROS-mediated diseases have been described, and several clinical studies have shown that endogenous depletion of antioxidant enzymes can be alleviated by exogenous antioxidants. The current interest in the use of exogenous antioxidants for the treatment of human diseases is leading to a better understanding of these diseases and facilitating the development of new therapeutics with antioxidant activity to improve their treatment. Consuming antioxidant-rich foods or taking antioxidant supplements reduces the risk of chronic diseases and promotes general well-being.

Overall, vitamins, polyphenols, and Se share parallels in their antioxidant metabolism pathways, mechanisms of action, and modulation of antioxidant enzyme activity. Both play crucial roles in maintaining cellular health and protecting against OS. However, their distinct chemical structures and metabolic pathways influence their bioavailability and potency, requiring careful consideration when assessing their potential health benefits.

## Figures and Tables

**Table 1:** Tocopherol compounds, according to R^1^, R^2^, and R^3^ substituents.

Structure	R^1^	R^2^	R^3^	Name
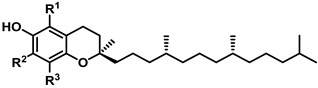	CH_3_	CH_3_	CH_3_	α-
	CH_3_	H	CH_3_	β-
	H	CH_3_	CH_3_	γ-
	H	H	CH_3_	δ-
